# Combined effects of *Rhodiola rosea* and caffeine supplementation on aerobic endurance and muscle explosiveness: a synergistic approach

**DOI:** 10.3389/fnut.2024.1335950

**Published:** 2024-03-13

**Authors:** Hezhang Yun, Bin Lu, Wenbo Su, Junjie Wang, Jing Zheng, Jing Wang, Zhaolong Wang, Yi Li, Yaowei Sun, Chang Liu

**Affiliations:** ^1^The Public Sports Department of the School, Zhejiang Guangsha Vocational and Technical University of Construction, Dongyang, China; ^2^School of Sport Science, Beijing Sport University, Beijing, China; ^3^School of Education, Philippine Women’s University, Manila, Philippines; ^4^Department of Sports Teaching and Research, Lanzhou University, Lanzhou, China; ^5^School of Military Civilian Integration Physical Training, Beijing Sport University, Beijing, China; ^6^State Key Laboratory of Toxicology and Medical Countermeasures, Beijing Institute of Pharmacology and Toxicology, Beijing, China; ^7^Yufeng Experimental School, Kunshan, China

**Keywords:** *Rhodiola rosea*, caffeine, muscle endurance, muscle explosiveness, erythropoietin, exercise performance

## Abstract

This study examined the synergistic effects of combining *Rhodiola rosea* (RHO) and caffeine (CAF) supplementation on muscle endurance and explosiveness in SD rats and human subjects, encompassing individuals without prior exercise training experience and seasoned aerobic athletes. Male SD rats and healthy human volunteers were randomly divided into four groups: CAF, RHO, CAF + RHO, and a control group (CTR). Nutritional supplements were administered throughout the training period, and pre-and post-measurement data were collected. In both the rat model and human subjects, the RHO+CAF group demonstrated significantly greater effects compared to the use of RHO or CAF supplements individually. Rats in the RHO+CAF group demonstrated extended running and swimming times and an increase in erythropoietin (EPO) mRNA expression in comparison to the CTR. Blood parameters, such as serum EPO levels, were enhanced in the CAF + RHO group, while blood urea nitrogen (BUN) and lactate (LA) levels significantly decreased in both the RHO and CAF + RHO groups. Hepatic and muscle glycogen contents were also higher in these groups. The gene expression analysis in rats demonstrated an elevation in the mRNA levels of glucose transporter-4 (GLUT-4), peroxisome proliferator-activated receptor γ coactivator-1 alpha (PGC-1α), Monocarboxylate transporter 1 (MCT-1), and Heme Oxygenase-1 (HO-1) in both the RHO and RHO+CAF groups. For individuals without prior aerobic training experience, the RHO+CAF group showed significant improvements compared to the CTR group in maximal oxygen consumption (VO_2_max), 5 km run, countermovement jump (CMJ), standing long jump, and 30 m sprint. For individuals with years of aerobic training experience, the RHO+CAF group exhibited enhanced performance in the 5 km run, CMJ, and standing long jump compared to the CTR group. In conclusion, the continuous 30 days supplementation of RHO, combined with a single dose of CAF, demonstrated superior effects on muscle endurance and explosiveness in both animal and human studies when compared to the use of RHO or CAF individually.

## 1 Introduction

As society’s emphasis on health and the pursuit of an improved quality of life steadily intensifies, there is a growing interest in the scientific exploration of strategies to augment athletic performance and physical adaptability. In this context, the utilization of natural herbal remedies has emerged as a pivotal area of research. Among these, RHO is a typical traditional herb renowned for its various health-promoting effects (1). Research reports suggest that supplementing with RHO positively impacts exercise capacity and performance without apparent adverse reactions (2). Participants taking RHO experience reduced pain and muscle damage after exercise training, improved skeletal muscle injury, enhanced antioxidant capacity leading to reduced oxidative stress, lower Ratings of Perceived Exertion (RPE) scores, and improved explosive strength, all without a decrease in perceived fatigue (2). Its primary component, rhodioloside (also known as salidroside), appears to effectively reduce fatigue and enhance athletic performance (1). Increasingly, researchers are focusing on the potential of natural supplements to enhance exercise performance and physiological adaptability. Within this domain, particular attention has been directed toward the utilization of *Rhodiola rosea* (RHO) and caffeine (CAF) as natural supplements, given their well-recognized roles in augmenting both muscle endurance and explosiveness (3–6). RHO holds a traditional place as a Tibetan medicine in China, celebrated for its remarkable vitality and distinctive adaptability. A key constituent of this herb is RHO glycoside, which is present in virtually all species within the RHO genus (7). Extensive research has underscored the fatigue-combating and exercise-amplifying properties of RHO glycoside (8–10). Some of these studies attribute these effects to its regulation of the hypoxia-inducible factor-1 (HIF-1) signaling pathway. HIF-1, functioning as a pivotal transcription factor, triggers the expression of erythropoietin (EPO) genes in hypoxic conditions, thereby exerting a significant influence on red blood cell production and oxygen transportation (1, 11, 12). Research also indicates that RHO supplements can enhance participants’ explosive resistance training performance (13). Continuous intake of RHO for 4 weeks can improve endurance exercise performance while also increasing VO_2_peak. Furthermore, studies suggest that oral administration of RHO extract can extend exhaustive swimming time in rats, activate the synthesis or resynthesis of ATP in mitochondria, and stimulate the energy repair process after intense exercise (7). Additionally, RHO has been shown to alleviate the level of fatigue after strenuous physical exercise, increase the concentrations of norepinephrine, dopamine, and serotonin in the brain, and elevate antioxidant levels in the plasma of professional athletes by reducing erythrocyte superoxide dismutase activity1,2. Professional athletes use RHO to enhance physical activity, stimulate skeletal muscle synthesis metabolism, increase endurance during maximal physical exercise, and promote subsequent recovery of the cardiovascular system (7, 14).

Caffeine, a naturally occurring stimulant abundant in coffee, has long been a subject of research and consistently demonstrates a strong connection with improved exercise performance (15). Acting as an antagonist to adenosine receptors, CAF boosts the release of neurotransmitters, notably dopamine and catecholamines (epinephrine and norepinephrine), leading to heightened alertness and physical prowess (16, 17). CAF possesses the capability to enhance neurotransmitter release. Multiple studies have affirmed that moderate CAF consumption can enhance both anaerobic and aerobic exercise capacities, including muscle endurance, speed, and explosiveness (16–18). Notably, despite its past classification as a prohibited substance, the World Anti-Doping Agency (WADA) subsequently removed CAF from the list of banned substances, rendering it a popular choice among athletes and fitness enthusiasts aiming to elevate their exercise performance.

However, despite extensive individual investigations into the potential effects of RHO and CAF, our understanding of whether their combined application can yield even greater enhancements remains relatively limited. The AMPK/Sirt1/PGC-1α pathway is considered a key regulatory factor in mitochondrial biogenesis (19, 20). Salidroside modulates mitochondrial biogenesis through the Sirt1/PGC-1α axis. 2PGC-1α, a downstream component of the AMPK pathway (21), plays a central role in regulating mitochondrial biogenesis, potentially influencing cellular energy metabolism and muscle endurance. Salidroside’s impact on this pathway suggests a potential influence on energy metabolism and muscle endurance. Additionally, RHO is believed to promote the synthesis or resynthesis of adenosine triphosphate (ATP) within mitochondria after high-intensity exercise and stimulate the energy recovery process, contributing to improved muscle function (7). CAF, on the other hand, enhances exercise performance by antagonizing adenosine receptors, leading to increased neurotransmitter release (22). Thus, we propose a hypothesis: co-supplementation of RHO and CAF may potentially generate synergistic effects, further augmenting muscle endurance and explosiveness.

The primary objective of this study is to investigate the potential synergistic effects of RHO and CAF supplementation on muscle endurance and explosiveness. By employing a comprehensive approach encompassing both a rat model and age-matched human participants, we aim to ascertain whether the combined application produces superior outcomes compared to individual supplementation. The exploration of these synergistic effects holds considerable significance for athletes and the broader population, offering valuable insights into the pursuit of safe and effective strategies to optimize exercise performance and physiological adaptability.

## 2 Materials and methods

### 2.1 Experimental subjects

#### 2.1.1 Animals

Sprague–Dawley (SD) rats were exclusively male, aged 8–9 weeks, with a weight of 200 ± 10 g. Male SD rats were randomly divided into four groups, each consisting of 12 rats: the CTR group, CAF group, RHO group, and RHO+CAF group. These rats were sourced from the Experimental Animal Science Department of Peking University and were housed in pairs in cages. The animal facility maintained a room temperature of 25 ± 1°C, with a relative humidity between 50 and 60%. The rats were subjected to a 12 h light/dark cycle. All animal procedures adhered to the “Regulations for the Management of Laboratory Animals” and were conducted under the production certificate No. SCXK (Beijing) 2016-0010 and the usage permit No. SYXK (Beijing) 2016-0041.

#### 2.1.2 Human subjects

This study recruited students from Beijing Sport University as research participants, comprising 4 groups of individuals without prior aerobic training experience and 2 groups of trained individuals matched for age and body mass index (BMI). Specifically, the term “non-aerobically trained subjects” refers to individuals who had no previous aerobic training experience before being included in our study. Each group comprised 12 participants, resulting in a total of 72 participants. Among the 48 voluntary participants in the exercise training program, they were randomly assigned to 4 groups (*n* = 12): CTR Group, CAF Group, RHO Group, and RHO+CAF Group. Participant recruitment for the human trials commenced 3 months before the formal experiment. Inclusion criteria involved individuals with approximately 5 years of aerobic exercise experience, demonstrating a robust training background and athletic performance. Due to the limited number of individuals meeting the aerobic training criteria, we successfully recruited 24 eligible participants, who were randomly divided into two groups (*n* = 12 each) for the experiment: CTR Group and RHO+CAF Group. Detailed demographic information of the participants is provided in Table 1. All participants underwent a 30 days aerobic training program.

**Table 1:** Body characteristics of untrained and trained resistance exercise volunteers.

Body characteristics	Resistance exercise-untrained volunteers (*n* = 48)	Resistance exercise-trained volunteers (*n* = 24)
Age (years)	20.04 ± 1.44	21.08 ± 1.25
Height (cm)	174.85 ± 5.10	178.83 ± 3.69
Weight (kg)	69.30 ± 9.31	71.79 ± 6.05
BMI (kg·m^−2^)	22.60 ± 2.60	22.46 ± 1.98
Lung capacity (ml)	4358.90 ± 764.47	5656.63 ± 411.47
Training experience (years)	–	5.08 ± 1.25

Before the experiment, each participant completed a questionnaire using the IPAQ scale to assess their physical activity intensity, providing insight into their activity levels. Additionally, all participants read and signed an informed consent form, indicating their understanding and willingness to participate in the study. To uphold research integrity and comply with the ethical standards of Beijing Sport University, agreements and signed commitments were obtained from all participants 3 months prior to the formal human trials. Participants declared their abstinence from caffeine, tea, additional nutritional supplements, enhancers, or alcohol-containing products that could significantly impact their athletic performance for the 3 months leading up to the study. Furthermore, all participants were non-smokers and had no neuromuscular or musculoskeletal disorders that could impede their ability to engage in regular exercise. Participants in the untrained group had not undertaken professional aerobic exercise training for at least 1 year before the study. Additionally, none of the participants were taking antidepressant or stimulant medications. This study adhered to the principles of the Helsinki Declaration and received approval from the Sports Science Experiment Ethics Committee of Beijing Sport University (Ethics Committee No. 2023279H).

### 2.2 Drug preparation and intervention

CAF was administered using CAF capsules from Nutricost in UT, United States, with each capsule containing 200 mg of anhydrous CAF. The pharmaceutical excipients in the CAF capsules included rice flour and gelatin. Rats in the CAF group and RHO+CAF group were orally gavaged with CAF at a dose of 19.7 mg per kg. RHO was sourced from Beijing Tong Ren Tang Pharmaceutical in China, and the RHO capsules included pharmaceutical excipients such as starch and gelatin. Rats in the RHO group and RHO+CAF group were orally gavaged with RHO at a dose of 262.7 mg per kg. In the CTR group, neither RHO nor CAF was administered throughout the entire experimental period. In the RHO group, rats received RHO continuously for 30 days through oral gavage. In the CAF group, CAF was orally administered half an hour prior to the final test on the last day. In the RHO+CAF group, rats received RHO continuously for 30 days and were also orally administered CAF half an hour before the final test on the last day.

For human subjects, CAF capsules were taken at a concentration of 3 mg per kg of body weight. Regarding RHO, the recommended daily dosage was 2.4 g. Participants in the RHO group consumed RHO continuously for 30 days, with a requirement to take RHO on an empty stomach 30 min before breakfast and lunch, while ingesting empty CAF capsules to minimize the potential influence of the placebo effect. In the CTR group, CAF or RHO powder was removed from the capsules, and only empty capsules were ingested. To mitigate the placebo effect, the placebo group consumed an equivalent volume of water when taking empty CAF and RHO capsules. Similarly, in the CAF group, participants also ingested empty RHO capsules, and on the final day, they were orally administered CAF half an hour before the test. In the RHO+CAF group, participants consumed RHO capsules continuously for 30 days and were also orally administered CAF half an hour before the final test on the last day.

### 2.3 Treadmill training and testing protocol for rats

The rats underwent a 30 days treadmill running training program on a treadmill with a 0° incline, conducting training sessions three times per week. The duration and intensity of exercise progressively increased during the training period, as follows: Week 1: Rats ran at 12 m/min for 30 min; Week 2: Rats ran at 15 m/min for 30 min; Week 3: Rats ran at 18 m/min for 30 min; Week 4: Rats ran at a constant speed of 20 m/min for 30 min. Before each training session, a 5 min warm-up was administered at a speed of 8 m/min. To minimize stress without using electrical shocks, a sponge was placed near the proximal end of the treadmill to gently tap the rats’ tails. The exhaustion test assessed the rats’ aerobic endurance. Rats ran on the treadmill at 25 m/min until exhaustion, determined when the rat could no longer maintain the appropriate speed despite continuous manual prodding for 1 min. At this point, the rat was removed from the treadmill, and the total running time was recorded.

### 2.4 Swimming testing protocol for rats

The rat exhaustion test was used to evaluate the aerobic endurance of the rats. In this test, the rats were forced to swim placing in a water tank with a depth of 50 cm and a temperature of 30°C. The load was adjusted by attaching lead weights to the tail of the animal, which was approximately 8% of the animal’s body weight. Exhaustion was determined when the rat did not surface within 8 s after sinking, and the exhaustion time was recorded.

### 2.5 Analysis of EPO gene expression in rat kidney tissue

Rats were anesthetized with intraperitoneal injection of pentobarbital sodium (50 mg per kg of body weight) immediately following the last exercise session. Subsequently, blood samples were collected from the rats, and euthanasia was performed. Tissues including kidneys, liver and skeletal muscles were collected for the follow-up tests.

Approximately 10 mg of renal cortex tissue were homogenized in 1 mL of Trizol reagent for extracting total ribonucleic acid (RNA) according to the manufacturer’s protocol. RNA concentration was quantified using a Nanodrop spectrophotometer (NanoDrop ND-1000, Thermo Fisher Scientific Inc., Waltham, Massachusetts, United States). One thousand nanograms of RNA were reverse-transcribed into cDNA using the High-Capacity cDNA Reverse Transcription Kit (Thermo Fisher Scientific Inc., Waltham, Massachusetts, United States). The specific primer sequences used were as follows: Forward primer for EPO (5′-3′): TACGTAGCCTCACTTCACTGCTT; Reverse primer for EPO (3′-5′): GCAGAAAGTATCGTGTGAGTGTTC; Forward primer for β-actin (5′-3′): CTTTCTACAATGAGCTGCGTG; Reverse primer for β-actin (3′-5′): TCATGAGGTAGTCTGTCAGG. Quantitative real-time polymerase chain reaction (qPCR) was conducted using the RT-qPCR reagent kit (Thermo Fisher Scientific, Waltham, Massachusetts, United States) and the StepOne Plus real-time fluorescence quantitative PCR system (Thermo Fisher Scientific, Waltham, Massachusetts, United States). The reaction involved an initial preheating step at 95°C for 10 min, followed by 40 cycles of denaturation at 95°C for 20 s, annealing at 60°C for 20 s, and extension at 72°C for 20 s. qPCR data were quantified based on the cycle number at which fluorescence reached a specific detection threshold (Ct value). The relative expression levels of genes were determined based on equal amounts of RNA (1 μg) and the average Ct value for each gene. β-actin served as the internal reference gene. Delta Ct (ΔCt = Ct_target gene_ − Ct_reference gene_) was calculated within the same sample. ΔΔCt was calculated as ΔΔCt = (ΔCt_treated_ − ΔCt_untreated_). The standardized expression changes were represented as 2^−ΔΔCt^, with β-actin control set to 100.

### 2.6 Enzyme-linked immunosorbent assay for serum erythropoietin

The collected rat blood samples were allowed to clot at room temperature for 30 min, followed by centrifugation at 12,000 rpm for 15 min to obtain serum samples. The concentration of EPO in the serum was determined using an ELISA with a standard EPO ELISA kit provided by Cusabio Biotech Co., Ltd., Wuhan, China. This assay utilized a quantitative sandwich enzyme immunoassay technique. In this method, EPO in the sample or standards was captured between pre-coated EPO antibodies and biotinylated EPO antibodies. The resulting antigen–antibody–antigen complex was subsequently labeled with horseradish peroxidase. After the removal of unbound reagents through washing, a substrate solution containing 3,3′,5,5′-tetramethylbenzidine was added to the wells. The color development that followed was directly proportional to the amount of EPO initially bound in the initial step. The color development was halted before measuring the color intensity using a microplate reader. Serum samples were maintained at −80°C until analysis, and all procedures were rigorously carried out in accordance with the manufacturer’s instructions.

### 2.7 Serum analysis in rats

After anesthetizing the rats, blood collection was performed, and the collected blood samples were stored at 4°C. They were then centrifuged in batches at a speed of 3,000 rpm for 15 min. The upper-layer serum was used for biochemical analysis to measure blood lactate (LA) and blood urea nitrogen (BUN) levels in the rats.

### 2.8 Glycogen assay in rats

The collected liver and skeletal muscle tissues were minced and homogenized on ice to prepare a homogenate. The supernatant was collected after centrifugation at 3,000 rpm for 15 min. The glycogen content in both liver and muscle tissues of each rat group was determined using the anthrone-sulfuric acid colorimetric method, following the manufacturer’s instructions. The reagent kit was obtained from Nanjing Jiancheng Bioengineering Institute, Nanjing City, China.

### 2.9 Rat RT-qPCR analysis

RT-qPCR was performed to measure the mRNA levels of peroxisome proliferator-activated receptor γ coactivator-1 alpha (PGC-1α), glucose transporter-4 (GLUT-4), monocarboxylate transporter 1 (MCT-1) and heme oxygenase-1 (HO-1) in skeletal muscle tissues. Total RNA was extracted from skeletal muscle tissues using Trizol, and cDNA was synthesized from the total RNA using a reverse transcription kit. qPCR was carried out using TB Green Fast qPCR Mix. Using the following specific primer sequences, quantitative real-time polymerase chain reaction (qPCR) was conducted: The forward primer (5′-3′) for PGC-1α was GAGGACACGAGGAAAGGAAGACT, and the corresponding reverse primer (3′-5′) was ACTGGCTTGAATCTGTGGAAGAAC (23). For GLUT-4, the forward primer (5′-3′) was GTGTGGTCAATACCGTCTTCACG, and the reverse primer (3′-5′) was CCATTTTGCCCCTCAGTCATTC (24). In the case of HO-1, the forward primer (5′-3′) was ACCCCACCAAGTTCAAACAG, and the reverse primer (3′-5′) was GAGCAGGAAGGCGGTCTTAG (25). For MCT-1, the forward primer (5′-3′) was CTTGTGGCGTGATCCT, and the reverse primer (3′-5′) was GTTTCGGATGTCTCGGG (26). Finally, the forward primer (5′-3′) for β-actin was CTTTCTACAATGAGCTGCGTG, and its reverse primer (3′-5′) was TCATGAGGTAGTCTGTCAGG (1).

### 2.10 Training protocol for humans

The aerobic exercise intervention in this study was conducted using a power treadmill training period 30 days. Training sessions occurred three times a week, on alternate days, scheduled in the evening between 19:00 and 21:00. The specific training protocol was as follows: Warm-up: All participants commenced with a 3 min warm-up at 35% of their maximum heart rate (HRmax). Exercise Phase: Following the warm-up, participants adjusted the treadmill speed to attain a heart rate within the range of 60 to 70% of their HRmax. This heart rate range was maintained during the 35 min exercise session. Cool-down: After completing the exercise, participants engaged in relaxation and cool-down activities. To effectively monitor heart rate during the intervention, both groups wore heart rate monitors for real-time tracking. Heart rate fluctuations were continuously monitored during the sessions, allowing for necessary adjustments to exercise intensity.

### 2.11 Daily dietary intake recording

Throughout the study period, all participants, including those who did not undergo resistance training and those who did, were instructed to maintain their regular diets. To further regulate habitual dietary intake, various packaging options compliant with national food safety regulations were provided to the participants. Dietary intake during the experiment was recorded in kilocalories (kcal), and daily intake of carbohydrates, proteins, and fats was quantified by weight (g). It is noteworthy that neither CAF nor RHO had an impact on participants’ appetite or food intake. Furthermore, Participants were informed to continue their regular physical activities during the study period, while avoiding intense exercises, especially aerobic and explosive strength training. Daily activities were monitored through a WeChat group established by the experimenters, recording participants’ daily step count to document their routine activities. This ensured accuracy and the smooth progression of the experiment. Calculations for daily total energy intake were based on the standard reference from the National Nutrient Database provided by the United States Department of Agriculture.

### 2.12 Maximal oxygen consumption measurement

Prior to the VO_2_max test, the testing personnel took the following steps: Preheated the equipment. Conducted gas calibration to ensure the equipment’s proper functioning. Prepared the participants, who were equipped with Polar heart rate monitors. The test began with a 3 min rest period to record resting data. Subsequently, exercise intensity was incrementally increased every 3 min until exhaustion, following the well-established Bruce protocol. The Bruce protocol is widely used for the quantitative increment of exercise load and the assessment of cardiorespiratory fitness. It comprises 7 predefined stages, each characterized by progressively higher treadmill speed and incline. The details of the test protocol are presented in Table 2.

**Table 2:** Bruce treadmill protocol.

Test stages	Speed (km/h)	Incline (°)	Duration (min)
Rest	0	0	3 min
Stage 1	2.7	10	3 min
Stage 2	4.0	12	3 min
Stage 3	5.4	14	3 min
Stage 4	6.8	16	3 min
Stage 5	8.0	18	3 min
Stage 6	8.8	20	3 min
Stage 7	9.6	22	3 min

During the final 30 s of each stage, the participant’s heart rate and Rating of Perceived Exertion (RPE) score were recorded. A member of the testing team closely monitored the participant’s condition throughout the test to ensure safety. They provided necessary encouragement and ensured that the participant maintained a steady pace and rhythm to prevent early termination due to nervousness or irregular pacing. Positive reinforcement was employed to motivate participants and maximize their exercise potential, thereby ensuring test accuracy. Upon reaching exhaustion, the following data points were recorded: Participant’s heart rate, Subjective fatigue level, Duration of exercise.

Termination Criteria: The VO_2_max test could be terminated under the following conditions: Rating of Perceived Exertion (RPE) ≥ 18, Respiratory Exchange Ratio (RER) ≥ 1.10, Reaching 90% of maximum heart rate (HRmax = 220 − age). Achieving a plateau in oxygen consumption where there is a change of less than 2 mL/min/kg over 2 min. Terminating the test was considered reaching maximal oxygen consumption. Additionally, if a participant displayed symptoms such as difficulty breathing, distress, body swaying, or pallor, the test was immediately terminated to ensure participant safety.

### 2.13 5 km test

Following the completion of the VO_2_max test and a minimum 72 h rest period, participants underwent a 5 km running time trial while in a fasted state. During this trial, participants were instructed to complete the 5 km as quickly as possible. The trial was conducted on a treadmill set at a 1% incline, and participants had control over their running speed. To prevent pacing strategies during the experiment, participants were shielded from viewing certain controls (e.g., incline) and visual display information (e.g., running speed and time) through the use of a specially designed shield. Dietary intake in the 24 h preceding the exercise trials was assessed, and participants were instructed to maintain consistent dietary habits, with a focus on carbohydrate, fat, and protein content, prior to each exercise trial.

### 2.14 Countermovement jump test

The CMJ test is a widely employed assessment for evaluating an individual’s explosive lower limb power and lower body strength during vertical jumping. In this test, participants begin from a standing position, execute a rapid downward movement by flexing their knees and hips, and then immediately leap upward with maximal force. The specific protocol is as follows: Participants stand with feet shoulder-width apart, gaze forward, hands on hips, and position themselves at the center of a force plate. They maintain an upright and stable trunk. Upon receiving the command “jump” from the test administrator, they quickly perform a downward movement, squatting deeply, and then explosively jump upward to reach their maximum height. Throughout the entire motion, hand position remains unchanged, upper bodies are kept upright, and leg extension is maintained after takeoff, ensuring full extension of the hip, knee, and ankle joints without folding or leg retraction. After each jump, a one-minute recovery period is observed, and three valid trials are conducted. The primary measurement outcome is the CMJ height, typically recorded in centimeters.

### 2.15 Standing long jump test

The Standing Long Jump test, also known as the Broad Jump test, assesses an individual’s lower body explosive power and horizontal jumping ability. Here is the test procedure: Participants stand with feet together, toes slightly apart, and hands hanging naturally at their sides with palms facing inward. Upon hearing the command “jump” from the test administrator, they exert force simultaneously with both legs, bending their knees and rapidly propelling themselves forward and upward. The jump’s distance is measured from the take-off line to the closest point of contact on the landing. Each participant is given three attempts with a 30 s rest between each jump. The best result among the three jumps is recorded as the participant’s score. This test is commonly used to evaluate lower body strength and power, as well as an individual’s ability to generate horizontal jumping force.

### 2.16 30-meter sprint test

The 30-Meter Sprint Test is conducted as follows: Prior to the sprint test, participants engage in a standard 15 min warm-up, involving activities such as easy jogging, stretching, and jumping exercises to prepare their muscles and cardiovascular system. After the warm-up, participants perform two maximal effort 30-meter sprints, exerting their best effort by sprinting as fast as possible for the entire 30 meters. The best time achieved among the two sprint attempts is recorded for data analysis. A mandatory 3 min rest period is observed between the two sprint attempts to allow participants adequate recovery time. This test evaluates an individual’s speed and acceleration over a short distance and is commonly used to assess sprinting capabilities.

### 2.17 Statistics

All results are presented as mean ± standard deviation (SD). Unpaired two-tailed *t*-tests were used for pairwise comparisons between two groups, while ordinary one-way ANOVA followed by Dunnett’s *post hoc* test was employed for comparisons involving multiple groups. Significance levels were set at *p* < 0.05 and *p* < 0.01 with “ns” indicating non-significance. Statistical analyses were performed using GraphPad Prism 9 and SPSS 22.0 software.

## 3 Results

### 3.1 Synergistic effects of RHO and CAF on rats

#### 3.1.1 Analysis of the expression of the rat EPO gene in rat kidney tissues, serum erythropoietin detection, and aerobic exercise results

**Figure 1:**
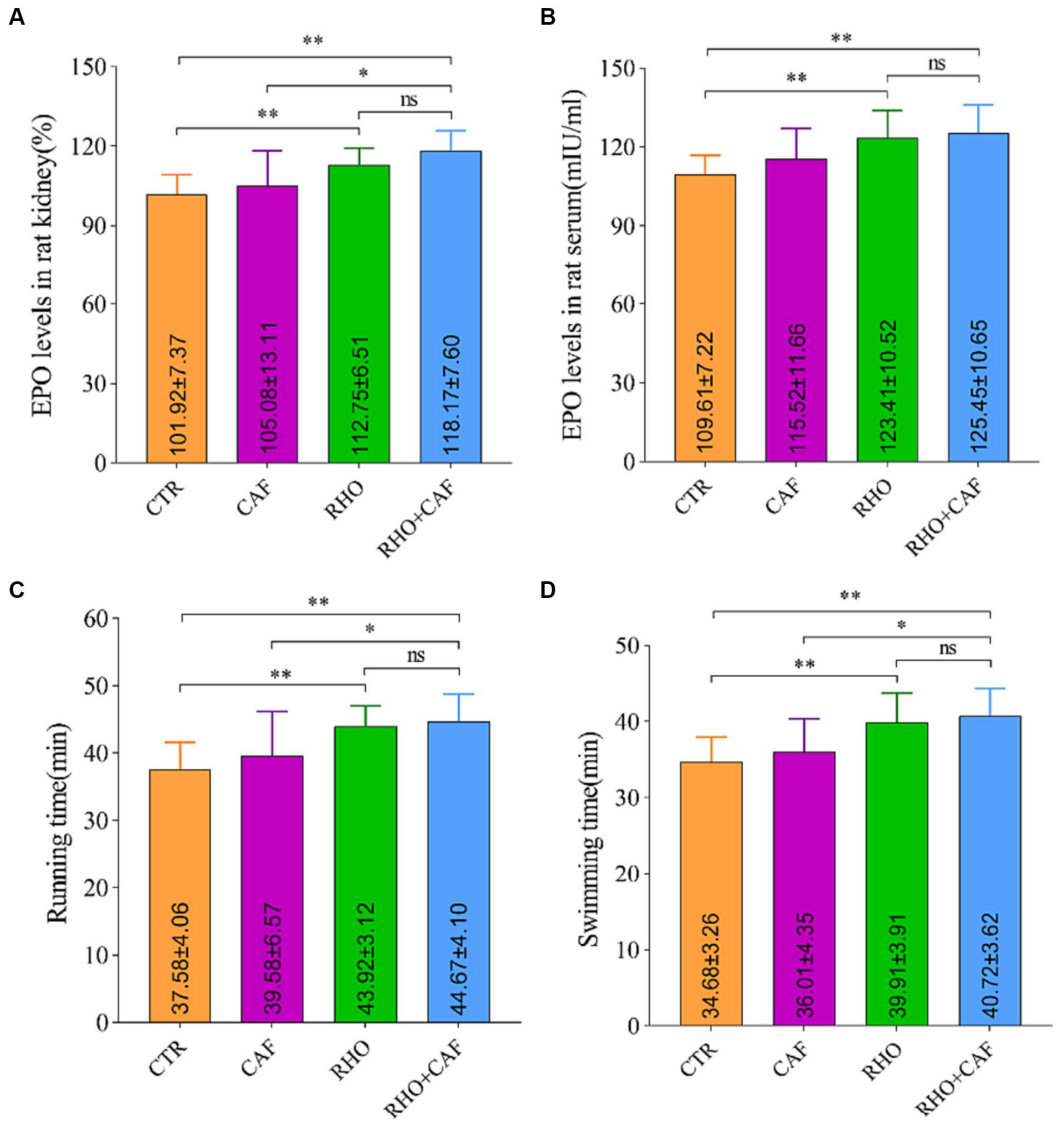
Analysis of the expression of the rat EPO gene in rat kidney tissues, serum erythropoietin detection, and aerobic exercise results. **(A)** Expression of the EPO gene in the rat kidney. **(B)** Serum erythropoietin levels. **(C)** Running time. **(D)** Swimming time.

To investigate the impact of CAF, RHO, and their combination on the expression levels of the EPO gene in rat kidney tissue, we employed quantitative real-time polymerase chain reaction (qPCR), using β-actin as an internal control. The results, illustrated in Figure 1A, reveal the following findings: In the CTR group, the EPO mRNA level in rat kidney tissue was measured at 101.92 ± 7.37. While the CAF group did not exhibit statistically significant differences compared to the CTR group, there was a slight increase in EPO mRNA expression observed in the CAF group. In contrast, both the RHO and CAF + RHO groups displayed significantly elevated levels of EPO mRNA in rat kidney tissue compared to the CTR group (*p* < 0.01). In this case, the elevation of EPO mRNA in rats from the CAF + RHO group showed a significant difference compared to the CAF group (*p* < 0.05). Notably, the RHO+CAF group demonstrated the most substantial increase in EPO mRNA levels, suggesting a more pronounced impact on EPO gene expression in rat kidney tissue.

The impact of different treatments on rat serum EPO levels was assessed using an ELISA assay kit (Figure 1B). The results yielded the following insights: In the CTR group, the baseline serum EPO level was measured at 109.61 ± 7.22 mIU/mL. Comparatively, the CAF, RHO, and RHO+CAF groups all exhibited elevated serum EPO levels in comparison to the CTR group. Of note, both the RHO and RHO+CAF groups demonstrated highly significant differences in serum EPO levels when compared to the CTR group (*p* < 0.01). These findings suggest that administering a single high dose of CAF in conjunction with 30 days of RHO rosea supplementation effectively increases serum EPO levels in rats.

We evaluated the effects of CAF, RHO, and their combination (CAF + RHO) on muscle endurance through the results of the treadmill experiment conducted on rats. The primary measure was the time rats spent running on a treadmill until exhaustion. As depicted in Figure 1C, supplementation with various exercise supplements led to an increase in running time for the rats. Here are the specific findings: In the CTR group, rats ran until exhaustion for an average duration of 37.58 ± 4.06 min. The CAF group of rats exhibited a longer running time, with an average of 39.58 ± 6.57 min. However, this difference was not statistically significant (*p* > 0.05) when compared to the CTR group. Conversely, both the RHO group and the CAF + RHO group of rats demonstrated significantly longer running times compared to the CTR group, with a statistically significant difference (*p* < 0.01) observed in both cases. Among them, the running time of rats in the CAF + RHO group showed a significant difference compared to the CAF group (*p* < 0.05). Notably, the RHO+CAF group effectively improved the muscle endurance of the rats.

The results of the rat swimming experiment aimed to assess the effects of CAF, RHO, and their combination (CAF + RHO) on rat muscular endurance by measuring the time they swam until exhaustion. As depicted in Figure 1D, the experimental results yielded the following observations: The CTR group of rats exhibited a swimming duration of 34.68 ± 3.26 min. On the other hand, the CAF group showed a modest increase in swimming duration compared to the CTR group. However, this increase was not statistically significant. In comparison to the CTR group, both the RHO and CAF + RHO groups of rats exhibited significantly prolonged swimming durations, showing highly significant differences in both cases (*p* < 0.01). Specifically, the swimming time of rats in the CAF + RHO group significantly exceeded that of the CAF group, indicating a significant difference (*p* < 0.05). It is noteworthy that the RHO+CAF group demonstrated the most substantial improvement in rat muscular endurance.

#### 3.1.2 Serum analysis and glycogen detection in rats

**Figure 2:**
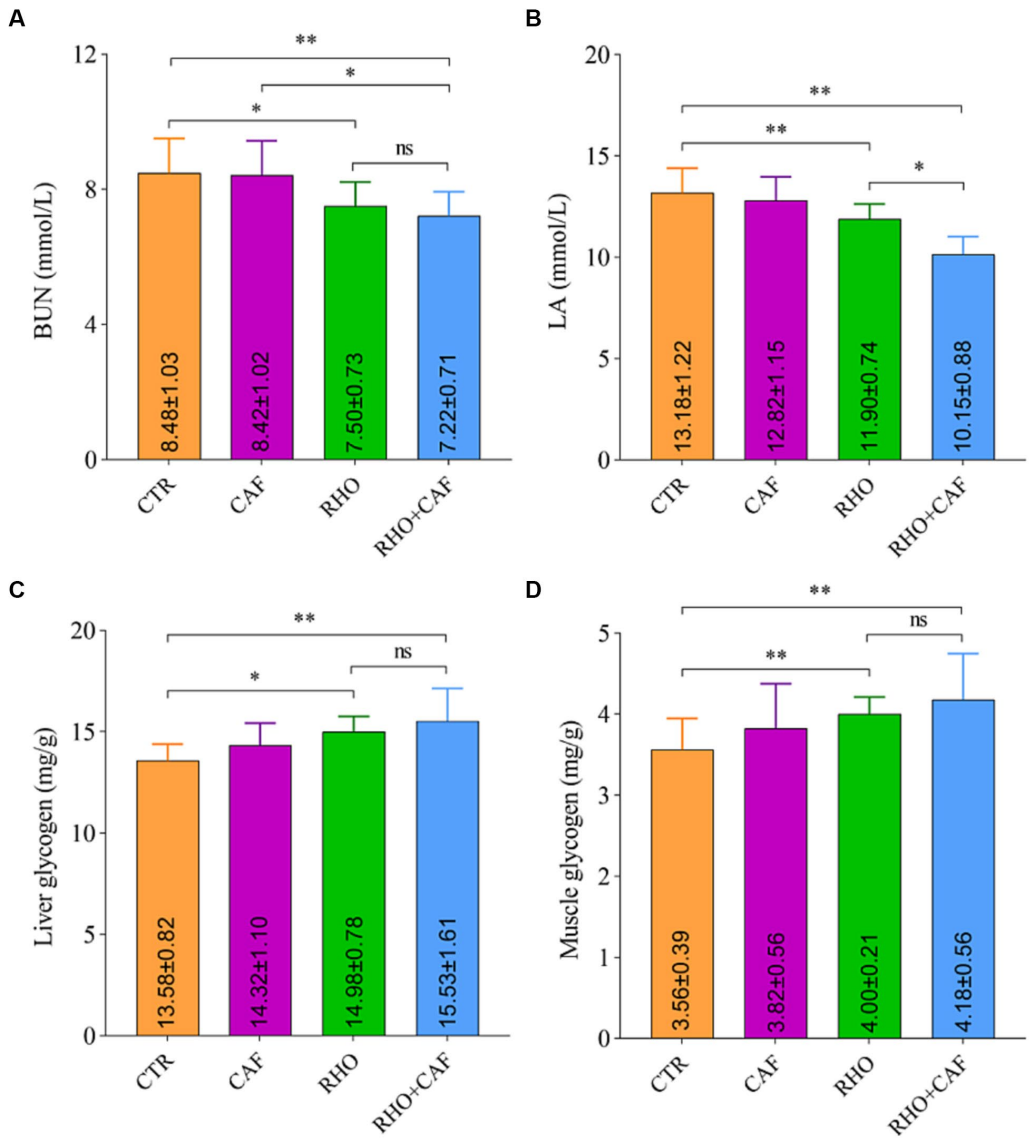
Serum analysis and glycogen detection in rats. **(A)** BUN. **(B)** LA. **(C)** Liver glycogen. **(D)** Muscle glycogen.

The results of the rat serum analysis and glycogen detection experiment under the influence of different treatments are shown in Figures 2A,B, indicating that the supplementation of various exercise supplements led to corresponding changes in rat blood BUN and LA levels. In the CTR group, the average blood BUN and LA levels in rats were the highest, measuring 8.48 ± 1.03 mmol/L and 13.18 ± 1.22 mmol/L, respectively. Notably, both the RHO group and the CAF + RHO group of rats exhibited a highly significant reduction in these two blood parameters when compared to the CTR group (*p*<0.05). In this case, compared to the CAF group, rats in the CAF + RHO group showed a significant decrease in BUN (*p* < 0.05); compared to the RHO group, rats in the CAF + RHO group exhibited a significant reduction in LA (*p* < 0.05). It is worth mentioning that the RHO+CAF group effectively improved rat muscle endurance, facilitating the faster degradation of BUN and LA in rat blood. This, in turn, shortened the recovery time from fatigue and enhanced the rats’ fatigue resistance.

Glycogen is a vital source of energy for the body, with higher levels of liver glycogen and muscle glycogen providing more energy reserves. As shown in Figures 2C,D, the CTR group of rats had the lowest levels of liver glycogen and muscle glycogen, exhibiting a highly significant difference compared to both the RHO group and the CAF + RHO group (*p* < 0.05). Importantly, the supplementation of CAF + RHO significantly increased the levels of both liver glycogen and muscle glycogen, further enhancing their fatigue resistance capacity.

#### 3.1.3 mRNA detection results in rat muscle tissues

**Figure 3:**
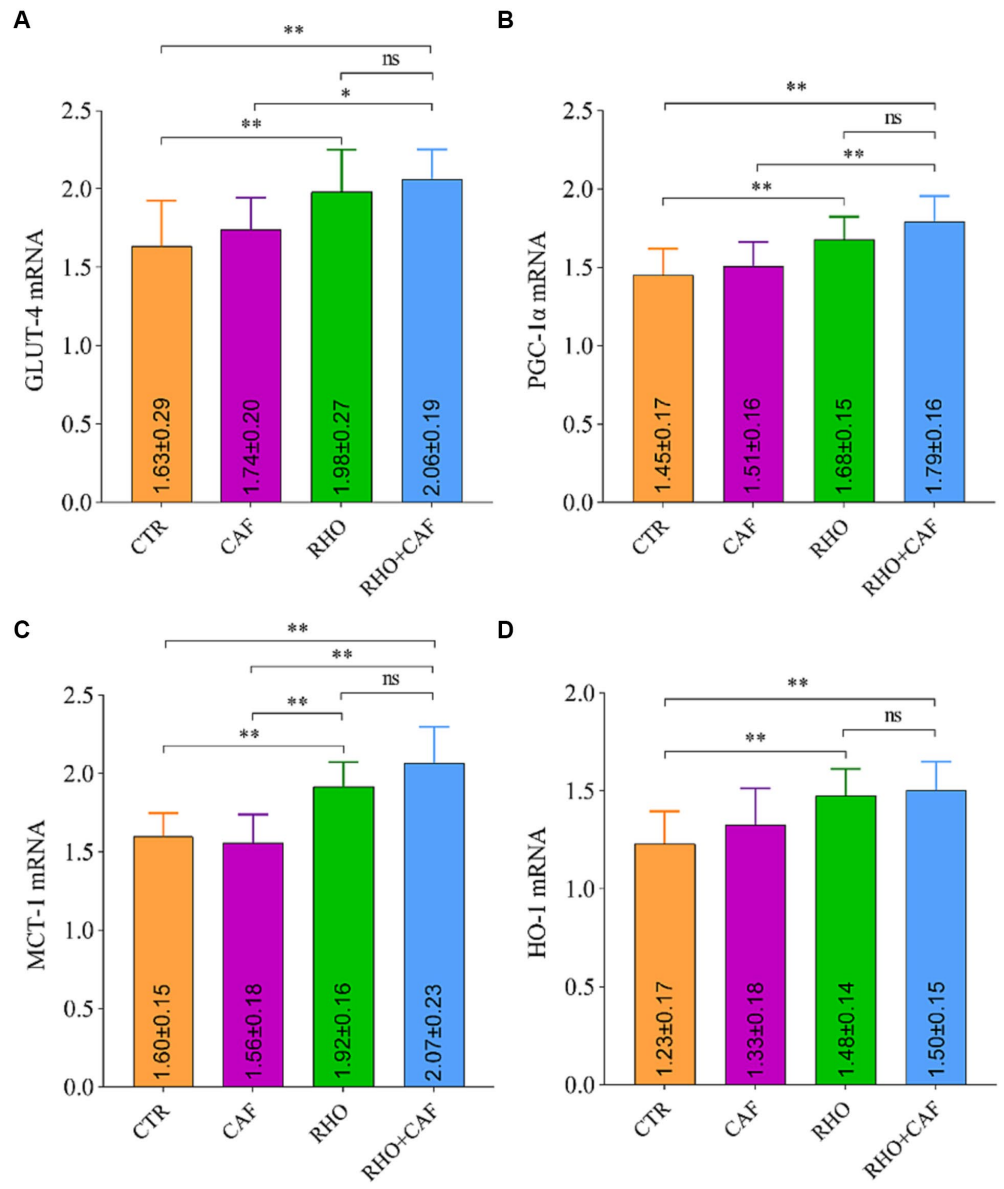
mRNA detection results in rat muscle tissues. **(A)** GLUT-4. **(B)** PGC-1α. **(C)** MCT-1. **(D)** HO-1.

The rat RT-qPCR test results are presented in Figure 3, where it is evident that both the RHO group and the RHO+CAF group exhibited significantly higher mRNA expression levels of GLUT-4 (Figure 3A), PGC-1α (Figure 3B), MCT-1 (Figure 3C), and HO-1 (Figure 3D) compared to the CAF and CTR groups. These differences were highly significant (*p*<0.01) in both cases. For the mRNA expression levels of GLUT-4, PGC-1α, and MCT-1, the RHO+CAF group exhibited higher expression compared to the CAF group, and these differences were statistically significant (*p* < 0.05). Specifically, the mRNA expression of MCT-1 also showed a statistically significant difference between the CAF and RHO groups (*p* < 0.01). Notably, the RHO+CAF group displayed the highest expression levels among the mentioned indicators.

### 3.2 Rhodiola and caffeine’s synergistic effects on human subjects

#### 3.2.1 Daily dietary intake records

**Figure 4:**
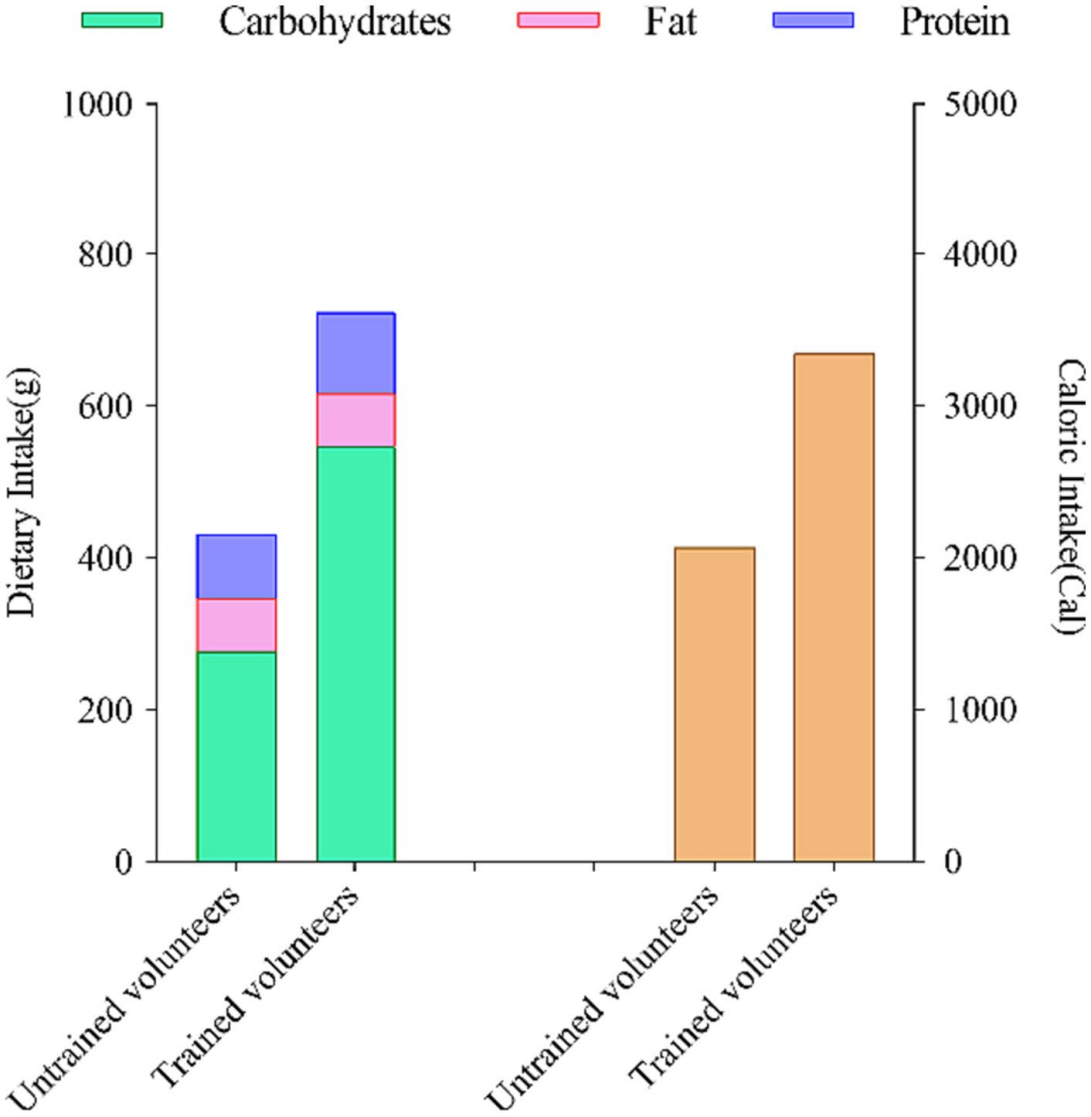
Dietary nutrient intake and composition of subjects in the 24 hours prior to two assessments.

To ensure consistency and minimize the potential impact of dietary factors, we provided each untrained and aerobically trained subject with a selection of fixed meal options. These meals were tailored to meet specific nutritional requirements. Here is a breakdown of the daily dietary intake records for both groups: For untrained volunteers, the dietary intake consisted of approximately 277 g of carbohydrates, approximately 85 g of protein, approximately 69 g of fat, resulting in a total energy intake of approximately 2069 kcal. In contrast, subjects with aerobic training experience had a dietary intake comprising approximately 574 g of carbohydrates, approximately 107 g of protein, approximately 69 g of fat, with a total energy intake of approximately 3,345 kcal. Throughout the entire study duration, all participants adhered to their regular dietary habits. Figure 4 outlines the nutritional intake of subjects in the 24 h leading up to the two assessments.

#### 3.2.2 Changes in participants without aerobic training experience receiving supplements alongside training

**Figure 5:**
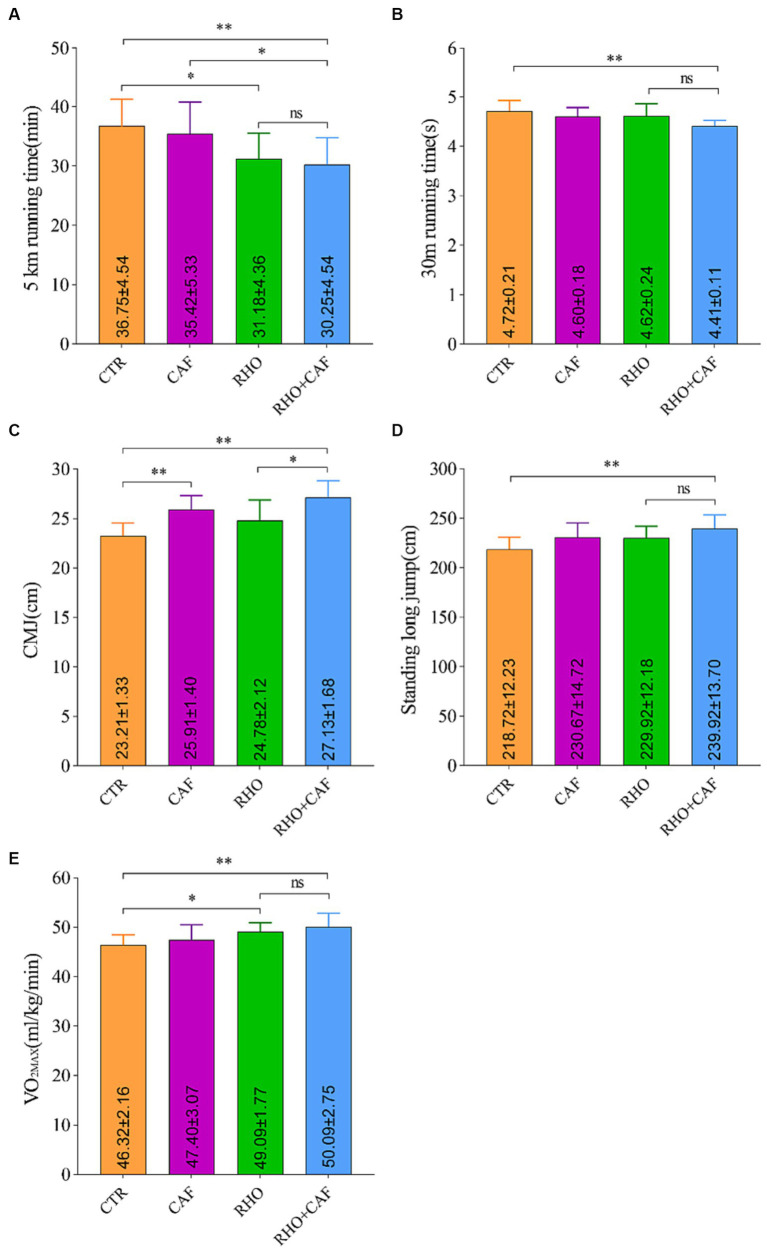
Exercise performance results of subjects without aerobic training. **(A)** 5 km running. **(B)** 30 m sprint. **(C)** Countermovement jump (CMJ). **(D)** Standing Long Jump. **(E)** Maximal oxygen consumption (VO2max).

In this study, we evaluated the aerobic performance of participants by measuring their 5 km running times under the influence of CAF, RHO, and CAF + RHO. The results, as depicted in Figure 5A, indicate the following: The 5 km running time for the Control (CTR) group was 36.75 ± 4.54 min. Both the CAF and RHO groups exhibited improvements in their 5 km running times compared to the CTR group. While the CAF group did not show statistically significant differences, there was a small improvement in their 5 km running times. Notably, both the RHO group and the CAF + RHO group displayed significant differences in their 5 km running times compared to the CTR group, with *p* values of <0.05 and < 0.01, respectively. Among them, the CAF + RHO group exhibited a significant reduction in the 5 km running time compared to the CAF group (*p* < 0.05). It is worth mentioning that the combination of RHO and a single high dose of CAF for 30 days further maximized the physical performance of participants without prior training experience.

The results of the 30 m Sprint Test for the participants are as follows (Figure 5B): In the CTR group, the 30 m sprint time was 4.72 ± 0.21 s. Both the RHO and CAF groups showed improvements in 30 m sprint performance compared to the CTR group. Although these improvements were not statistically significant, they indicated enhanced performance in the 30 m sprint. Importantly, both the CAF group and the CAF + RHO group displayed highly significant differences in 30 m sprint performance compared to the CTR group, with *p* values <0.01. The combination of RHO and a single high dose of CAF for 30 days further enhanced the physical performance of participants without prior training experience.

The CMJ test is a commonly used method to evaluate an individual’s explosive lower limb power and lower body strength in vertical jumping. Here are the results of the CMJ test (Figure 5C): In the CTR group, the CMJ score was 23.21 ± 1.33 cm. Both the CAF group and the CAF + RHO group exhibited highly significant differences in CMJ performance compared to the CTR group, with *p* < 0.01. Additionally, when comparing the RHO+CAF group to the RHO group, a significant difference in CMJ performance was observed, with *p* < 0.05. It’s worth noting that in participants without prior training experience, the combined application of RHO and a single high dose of CAF for 30 days further enhanced physical performance.

The results of the Standing Long Jump test for participants are as follows (Figure 5D): In the CTR group, the standing long jump score was 218.72 ± 12.23 cm. Both the RHO and CAF groups showed improvements in standing long jump performance compared to the CTR group. While these improvements were not statistically significant, they indicated enhanced performance in the standing long jump. Importantly, the CAF + RHO group displayed highly significant differences in standing long jump performance compared to the CTR group, with *p* values <0.01. It is noteworthy that the combined application of a 30 days regimen of RHO with a single dose of CAF further enhances their physical performance in participants without prior training experience.

To assess the aerobic exercise capacity of the participants, we conducted measurements of VO_2_max in response to CAF, RHO, and CAF + RHO supplementation (Figure 5E). The CTR group exhibited a VO_2_max of 46.32 ± 2.16 mL/kg/min. While the CAF group did not show a statistically significant difference compared to the CTR group. Both the RHO group and the CAF + RHO group showed significantly higher VO_2_max values with *p* < 0.05 and *p* < 0.01, respectively. Importantly, the combined use of 30 days of RHO with a single high dose of CAF further maximized the physical performance of untrained subjects.

#### 3.2.3 Improvements in participants with aerobic exercise training experience receiving supplements alongside training

**Figure 6:**
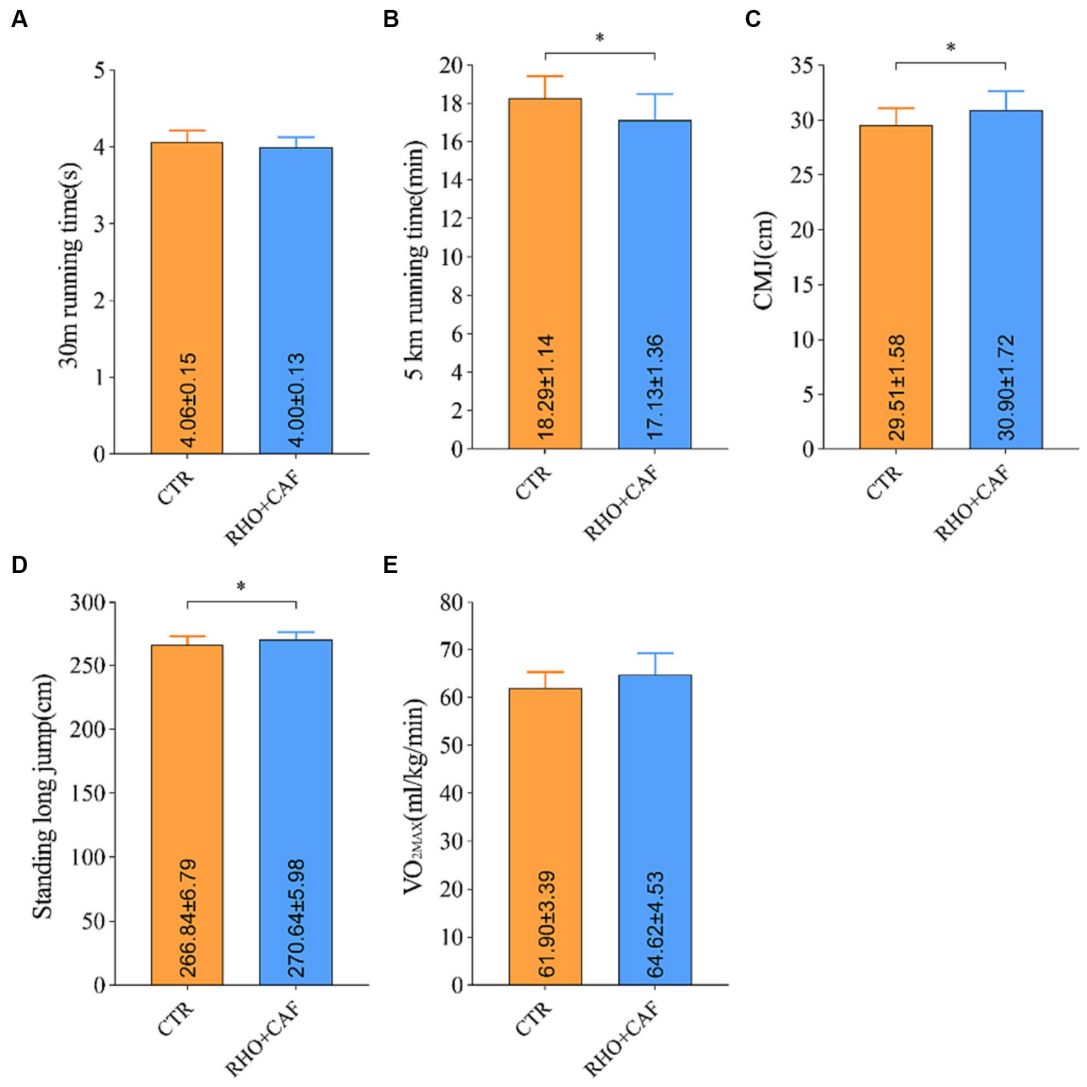
Improvement in performance metrics for aerobic exercise training volunteers following RHO+CAF supplementation. **(A)** 30 m sprint. **(B)** 5 km running. **(C)** Countermovement jump (CMJ). **(D)** Standing Long Jump. **(E)** Maximal oxygen consumption (VO2max).

Based on the results obtained from the aforementioned studies, it is evident that a 30 days regimen of RHO, coupled with acute CAF supplementation, yields superior enhancements in the physical performance of participants without prior training experience. This effect stands in contrast to the outcomes of exclusive RHO or CAF supplementation. Furthermore, the effectiveness of RHO+CAF supplementation was additionally assessed among participants with several years of aerobic training experience to evaluate its potential for enhancing physical performance. Recruited volunteers with years of aerobic training experience had an average training duration of 5.08 ± 1.25 years. As depicted in Figure 6, all test scores of the 12 aerobically trained participants exhibited significant improvements following RHO+CAF supplementation in comparison to their baseline performance and the CTR group. These improvements encompassed the 30 m sprint, CMJ, 5 km run, standing long jump and VO_2_max. With the exception of the 30 m sprint and VO_2_max, all other metrics demonstrated significant differences compared to the CTR group, with *p* values <0.05. These findings collectively suggest that a 30 days regimen of RHO in conjunction with acute CAF supplementation is effective in enhancing the athletic performance of individuals with several years of training experience.

## 4 Discussion

We conducted a study to assess the impact of different supplements on various performance metrics. The comprehensive findings of this research indicate that the combined supplementation of RHO and CAF has a positive effect on athletic performance, observed not only in animals but also in humans. In order to investigate the synergistic effects of RHO and CAF on muscle endurance and explosive power, we utilized SD rats and human subjects as participants in the study. Proper doses of RHO and CAF were administered during a 30 days period of running training. The results demonstrate that the RHO+CAF group exhibited significant improvements across various parameters, including muscle endurance and explosive power, compared to the CTR group. Additionally, the continuous supplementation of RHO alone for 30 days also showed significant improvements in muscle endurance for both humans and rats. Previous studies have suggested a potential link between RHO and improved muscle endurance through its ability to increase erythropoietin (EPO) expression (1, 11, 12). Therefore, in this study, we examined the gene expression levels of the EPO gene in rat kidney tissue and serum. The research findings indicate that the combined use of RHO and CAF effectively upregulates EPO expression. In the RHO+CAF group of rats, kidney EPO and serum EPO increased by 4.81 and 1.65%, respectively, compared to the RHO group. This increase in EPO expression is a crucial factor in enhancing athletic performance, consistent with our previous research results (1). Furthermore, continuous supplementation of RHO also demonstrated a significant increase in the oxygen consumption rate in muscle fibers, a key indicator of skeletal muscle mitochondrial respiration. This improvement suggests a corresponding increase in mitochondrial oxidative phosphorylation activity, contributing to enhanced physical performance, particularly in aerobic exercises. These findings align with previous research results (1, 27). Regarding aerobic exercise capacity indicators, such as rat running time, rat swimming time, and human subjects’ 5 km run performance and VO_2_max, the RHO+CAF group showed varying degrees of growth compared to the RHO group, with increases of 1.71, 2.03, 2.98, and 2.04%, respectively. This reflects to some extent the synergistic effect of the combined use of RHO and CAF. Additionally, the levels of oxidative stress response substances in skeletal muscles play a crucial role in regulating muscle function (28). RHO supplementation has been considered a positive strategy for preventing oxidative stress and enhancing exercise performance. Its extract can enhance antioxidant capacity by upregulating antioxidant enzymes, reduce skeletal muscle damage, and enhance the body’s anti-fatigue ability (14, 29, 30). Similarly, previous research suggests that individuals taking RHO may experience alleviated pain and muscle damage after exercise training, leading to improved athletic performance (2, 27).

In this study, while individual supplementation of RHO alone demonstrated an improvement in muscle endurance for both humans and rats, a significant difference in blood lactate clearance rate emerged between the RHO+CAF group and the RHO group following rat exercise. The lactate clearance rate in the RHO+CAF group was faster than that in the RHO group (*p* < 0.05) (Figure 2B). Animal experiments have indicated that supplementing with RHO can lead to varying degrees of reduced lactate levels, increased hepatic and muscular glycogen content, and enhanced ATPase activity (31). Intake of CAF has been proven to significantly elevate plasma levels of free fatty acids (FFA) and glycerol before exercise (32). The accumulation of FFA is known to reduce the rate of glycolysis by inhibiting phosphofructokinase. Therefore, it can be anticipated that pre-exercise CAF consumption may alter carbohydrate metabolism, consequently affecting the cellular lactate production rate (33). In our research, while both the RHO+CAF group and the RHO group showed significant differences compared to the CTR group, there was also a significant difference between the RHO+CAF group and the RHO group, with the lactate clearance rate in the RHO+CAF group being faster by 14.71%, suggesting a synergistic effect of the combined use of RHO and CAF. Similarly, in the human subjects’ explosive power as indicated by the CMJ, the RHO+CAF group and the RHO group also showed significant differences (*p* < 0.05) (Figure 5C). In this study, the supplementation of a certain dose of RHO alone did not show significant differences in lower limb explosive power indicators, such as standing long jump, CMJ, and 30 m sprint performance, compared to the CTR group of subjects without prior aerobic exercise experience. However, we found that the combined use of RHO+CAF produced better results than using CAF or RHO alone. Compared to the control group, subjects in the RHO+CAF group exhibited significant differences in standing long jump, CMJ, and 30 m sprint performance (*p* < 0.05). Additionally, when comparing the RHO+CAF group to the RHO group, there was an increase of 4.35, 9.48, and 4.55% in the aforementioned indicators, respectively. One possible explanation is that while a single intake of RHO can significantly increase hemoglobin levels by upregulating EPO in the bloodstream, the body may not be efficiently mobilizing muscles for short bursts of explosive power to maximize its effects. Under the joint action of CAF, the body not only benefits from the significant increase in hemoglobin brought by RHO but also promotes the release of various neurotransmitters, maintains alertness, enhances neuromuscular recruitment capabilities, ultimately achieving a stronger performance boost compared to the RHO group.

Recent research indicates that AMP-activated protein kinase (AMPK) functions as an energy sensor, and its activation promotes glucose uptake while inhibiting fatty acid synthesis (34, 35). During physical activity, activated AMPK regulates glucose and lipid metabolism to meet the body’s energy demands (36). Enhanced expression of PGC-1α, a downstream component of the AMPK pathway, can boost muscle endurance (21). Hexokinase 2 (HK2) is a key enzyme in glucose metabolism and glycolysis. AMPK upregulates HK2 expression, increasing glucose uptake in skeletal muscle cells. Exercise also increases the expression of GLUT-4, facilitating glucose transport and utilization in skeletal muscle cells. MCT-1 is a regulator of lactate uptake, and its activity is regulated by PGC-1α. Elevated MCT-1 expression increases lactate uptake in skeletal muscle, helping control overall lactate levels during exercise. This study demonstrates that RHO increases the phosphorylation of AMPKα in fatigued rat skeletal muscles, along with elevated mRNA levels of PGC-1α, GLUT-4, and MCT-1. Supplementing with RHO accelerates lactate degradation, speeds up lactate clearance, and slows the accumulation of lactate, ensuring orderly recovery. It is noteworthy that the combined supplementation of CAF and RHO also has significant effects. Compared to the Rhodiola-only group, the RHO+CAF group showed varying degrees of increase in mRNA expression levels of PGC-1α, GLUT-4, and MCT-1, with increases of 6.55, 4.04, and 7.81%, respectively. Urea nitrogen (BUN) is a metabolic byproduct of protein metabolism and serves as an indicator of exercise intensity, positively correlating with fatigue. Protein metabolism increases during recovery and exercise periods. Data analysis reveals that RHO supplementation decreases serum BUN levels. By regulating BUN levels, RHO supplementation reduces the proportion of energy derived from protein, thereby enhancing the functionality of glycogen during exercise. This clearly illustrates that RHO significantly improves the body’s ability to clear BUN, strengthening its anti-fatigue effects. It is noteworthy that the combined supplementation of CAF and RHO also has significant effects. Compared to the RHO group, the RHO+CAF group showed varying degrees of improvement in serum BUN, hepatic glycogen, and muscular glycogen levels in rats, with improvements of 3.73, 3.67, and 4.50%, respectively. Nrf2 (nuclear factor erythroid 2-related factor 2) is a central transcription factor regulating endogenous antioxidant pathways, with many antioxidant enzymes under the control of Nrf2. Under normal conditions, Nrf2 remains inactive in the cytoplasm by binding to Kelch-like ECH-associated protein 1 (Keap1) (37). Under oxidative stress, Nrf2 dissociates from Keap1 and translocates to the nucleus, activating the transcription of genes involved in antioxidant responses, such as HO-1 (37, 38). Activation of the Nrf2 pathway contributes to fatigue delay. This study demonstrates that salidroside increases the mRNA levels of HO-1 in the skeletal muscles of rats, suggesting that the mechanism through which salidroside inhibits exercise-induced fatigue may be linked to the AMPK and Nrf2 pathways. In light of current research advancements, this study suggests that salidroside may serve as a multifunctional dietary supplement in sports nutrition.

Numerous studies have confirmed the performance-enhancing effects of CAF on muscle, especially on muscle endurance (39–41). On one hand, the direct stimulation to the central nervous system by CAF can lead to delayed fatigue, increased alertness, and attention (42). On the other hand, the structural similarity between CAF and adenosine leads to an antagonistic effect of CAF on adenosine receptors, thereby preventing adenosine from binding to A1 or A2a receptors. This antagonistic effect can conversely promotes the release of neurotransmitters (43), such as an increased release of dopamine in the striatum stimulated by CAF (1, 44). In practice, it was reported that the ingestion of 3 mg·kg^−1^ of CAF had a significant positive impact on recreational male runners’ 5 km race times (45). On the contrary, some studies suggested that the acute supplementation of 3 mg·kg^−1^ of CAF could not significantly improve certain athletic abilities in human subjects, such as CMJ, squat jump, 5–10 m sprint, running speed, and the quality of athlete technical movements (46–49). As indicated by the current results, the changes in various indicators with the sole use of CAF are quite limited. However, once individuals undergo 30 days of RHO supplementation, subsequent acute administration of CAF results in more significant changes. This suggests a synergistic effect between long-term RHO administration and single-dose CAF administration. Compared to the RHO group, RHO+CAF showed improvements on the tested indexes ranging from 1.35 to 14.71% in both animal and human subjects, unfortunately, most changes did not exhibit statistical significance. This could be attributed to two main reasons. Firstly, the acute supplementation dosage of single-dose CAF in this study was relatively low, with CAF supplementation for human subjects at only 3 mg·kg^−1^ body weight. Previous study showed that dosages of 3–9 mg·kg^−1^ CAF produced positive implications for endurance running performance and fatigue time in human subjects (41). Moreover, the general dosage used in most studies is 6 mg·kg^−1^ (15). Warren et al. investigated (50) the dose–response effect of CAF intake on its impact on muscle endurance and found that for each 1 mg·kg^−1^ increase in CAF, the relative effect value of muscle endurance increased by 0.10. However, given the dose-dependent side effects of CAF, such as CAF tolerance, restlessness, and gastrointestinal discomfort, et al., we did not attempt to use higher dosages to test whether statistically significant changes could be observed in the measured indexes. Actually, the dose threshold and therapeutic window of CAF for combined use (RHO+CAF) are worth being explored and optimized in further studies. Secondly, the inherent limitations of the current study may potentially impact the statistical significance of various indicators, such as a relatively small sample size and substantial individual differences among experimental samples/subjects. Additionally, neither RHO nor CAF are classified as dopes, thus RHO+CAF would not possible to cause excessive changes to the body in such a short period of time (within 30 days). However, for athletes, especially for professional athletes, any positive changes will help them gain advantages in the fierce competition to achieve breakthrough results. Although preliminary, the current work provides proof-of-concept evidence for improving physical performance by combined supplementation of RHO and CAF, safely and effectively.

The results above indicate that supplementation with RHO+CAF not only enhances physical performance in animals and human volunteers without prior aerobic training experience but also provides benefits to individuals with years of aerobic training. This nutritional supplementation proves effective in improving overall physical capabilities and enhancing sports performance. To validate the impact of combined RHO+CAF supplementation on athletic performance, 24 participants with extensive aerobic training experience were recruited. Overall, the combined supplementation of RHO+CAF led to improvements in the subjects’ standing long jump, CMJ, 30 m sprint, VO_2_max, and 5 km race performances. When compared to the CTR group, there were statistically significant increases in the subjects’ standing long jump, CMJ, and 5 km race performances. While the 30 m sprint and VO_2_max did not exhibit statistically significant differences compared to the CTR group, they showed noticeable trends of improvement.

## 5 Conclusion

In conclusion, the collective evidence from animal and human studies, considered from multiple aspects, indicates that the combined supplementation of RHO for 30 days along with a single dose of CAF holds promise in enhancing muscle endurance and explosive power. This combination appears to provide superior benefits compared to the use of RHO or CAF as standalone supplements.

## Data availability statement

The raw data supporting the conclusions of this article will be made available by the authors, without undue reservation.

## Ethics statement

The studies involving humans were approved by Institutional Review Board of Beijing Sport University. The studies were conducted in accordance with the local legislation and institutional requirements. The participants provided their written informed consent to participate in this study. The animal study was approved by Institutional Review Board of Beijing Sport University. The study was conducted in accordance with the local legislation and institutional requirements.

## Author contributions

HY: Conceptualization, Formal analysis, Investigation, Project administration, Supervision, Writing – original draft. BL: Methodology, Writing – original draft. WS: Investigation, Methodology, Supervision, Writing – review & editing. JuW: Visualization, Writing – review & editing. JZ: Data curation, Validation, Writing – original draft. JiW: Data curation, Formal analysis, Validation, Writing – original draft. ZW: Methodology, Writing – original draft. YL: Conceptualization, Investigation, Supervision, Writing – review & editing. YS: Conceptualization, Data curation, Writing – review & editing. CL: Conceptualization, Project administration, Supervision, Writing – review & editing.
